# A quantitative meta-analysis comparing cell models in perfused organ on a chip with static cell cultures

**DOI:** 10.1038/s41598-023-35043-5

**Published:** 2023-05-22

**Authors:** Martin Dufva

**Affiliations:** Department of Health Technology, Technical University of Denmark, 2800 Kgs Lyngby, Denmark

**Keywords:** Biomaterials, Biological models, Lab-on-a-chip

## Abstract

As many consider organ on a chip for better in vitro models, it is timely to extract quantitative data from the literature to compare responses of cells under flow in chips to corresponding static incubations. Of 2828 screened articles, 464 articles described flow for cell culture and 146 contained correct controls and quantified data. Analysis of 1718 ratios between biomarkers measured in cells under flow and static cultures showed that the in all cell types, many biomarkers were unregulated by flow and only some specific biomarkers responded strongly to flow. Biomarkers in cells from the blood vessels walls, the intestine, tumours, pancreatic island, and the liver reacted most strongly to flow. Only 26 biomarkers were analysed in at least two different articles for a given cell type. Of these, the CYP3A4 activity in CaCo2 cells and PXR mRNA levels in hepatocytes were induced more than two-fold by flow. Furthermore, the reproducibility between articles was low as 52 of 95 articles did not show the same response to flow for a given biomarker. Flow showed overall very little improvements in 2D cultures but a slight improvement in 3D cultures suggesting that high density cell culture may benefit from flow. In conclusion, the gains of perfusion are relatively modest, larger gains are linked to specific biomarkers in certain cell types.

## Introduction

The motivation for organ on a chip (OOC) is that it is believed to reproduce human physiology and human molecular biology in vitro better than traditional cell culture methodology^1–3^. A key driver for OOC is that experimental animals does not translate well to humans in drug development^4,5^.

**Figure 1:**
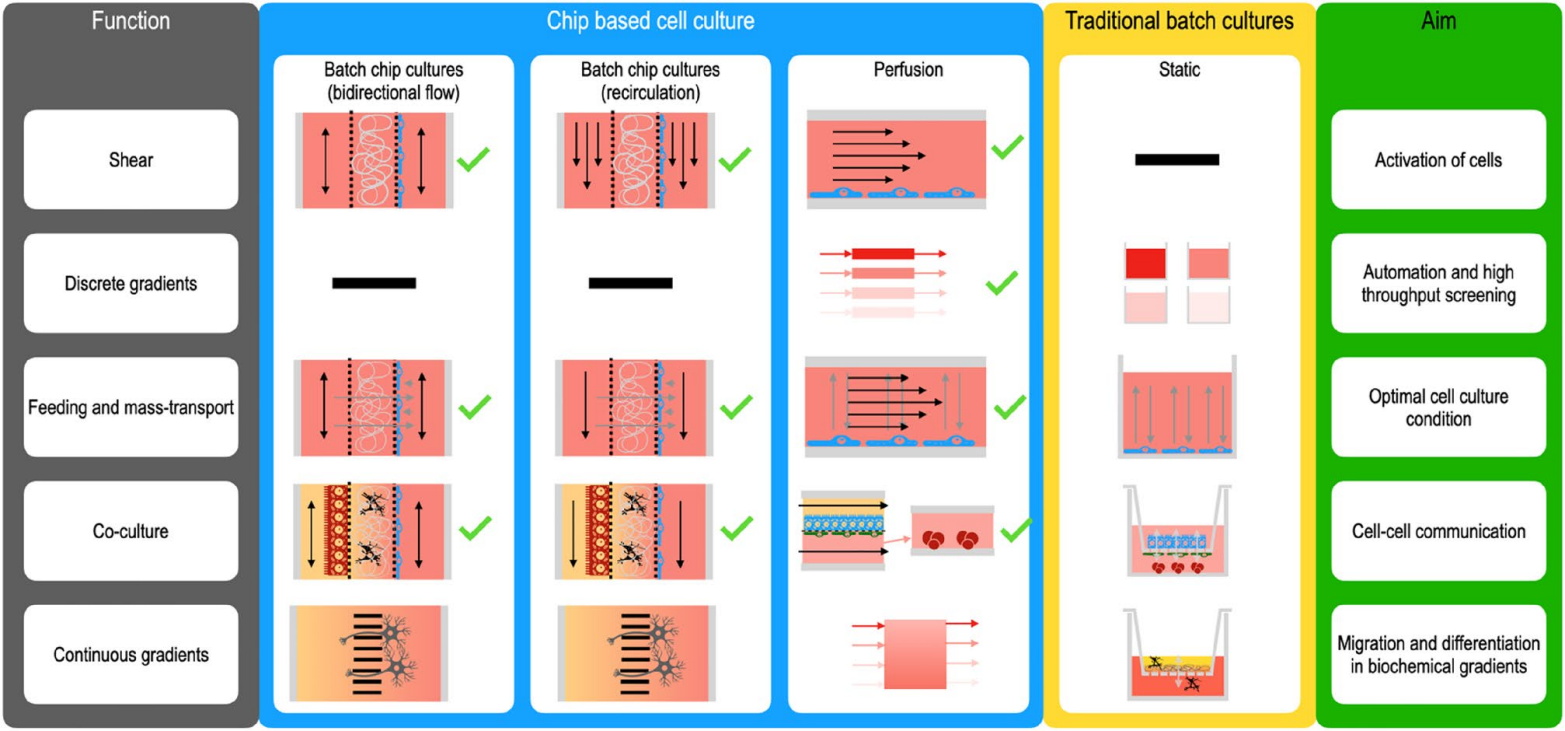
Overview of different cell culture methods. The green tick marks indicate which conditions that were included in the meta-analysis. Chips can be operated in many ways. The medium passes the cells only once in perfused chips. Alternatively, the medium is recirculated using either one directional or two directional flows. Recirculation is a form of batch culture with, normally, a larger medium to cell ratio than to wells and flasks. The traditional culture box indicates configurations to obtain similar functionalities as in the corresponding chips. Many of the plate/well controls could be agitated to provide better mass transfer (mixing) or shear but these controls were not generally used in the literature.

The traditional cell culture system (plates, flasks, Transwells, Fig. 1) is well known. It is modular, easy to use, standardised, aimable to automation and commercially available. Nearly all cell culture protocols and mediums compositions are developed for static cultures. While a well or plate seems like a trivial device, it can be modified with hydrogels enabling co-cultures and 3D cultures. It can also be modified with Transwell inserts^6^ to connect different cell types and to create barriers. The combination of hydrogels with Transwell inserts allows for triple cocultures to model the brain, intestinal absorption, or neuroprotective effects of mesenchymal stem cells, respectively^7–9^. The Transwell concept can be extended to multilayers or multi-compartments^10,11^ to connect more tissues. They are however still static in nature which affect mass transport and there is furthermore no shear. This may affect functions of cells as well as the kinetics of reactions. However, shear^12,13^ and mixing^11^, which typically are the strong points of microfluidics, have been demonstrated in plates using rocker systems or orbital shakers. Orbital shakers achieved > 10 dyne/cm^2^ at the periphery of a wide diameter well and aligned and activated endothelial cells^12,13^. Hence, using simple means, wells and plates can provide some features that often are associated with microfluidics such as interconnected tissues and in some cases shear and mixing. The fluidic control is however challenging, and some features cannot be done such as stable gradients over time for migrations studies (Fig. 1).

Organs on a chip (OOC) uses by contrast microfluidics solutions to provide physical cues such as shear and increased mass transfer which requires flow (Fig. 1). These chips may provide more relevant cell models than a simpler batch cultures^1,14^ but a broad *quantitative and data driven test* of this hypothesis is lacking in the literature despite numerous reviews of the field.

Microfluidic systems consist of a chip, a pumping mechanism and interconnections between the chip and the pumping mechanism. Therefore, these systems are complicated especially when reaching high degree of parallelisation^15,16^. Hence the gain of flow control comes with the price of complexity. There are several indications that flow is advantageous. For instance, endothelial cells are sensitive to shear stress with associated changes in morphology and molecular profile^17–19^. Intestinal epithelial cells are exposed to much lower shear but also that can induce changes in cell function like secretion of mucus or induction of 3D growth^20,21^. There are several chips that models the intestine^21^ and lung^22^. These chips are very similar to Transwell inserts as they contain a membrane that divides a larger flow chamber into an upper and lower chamber that are individually perfused (Fig. 1). The chips are either made entirely of PDMS^20^ or using a Transwell type of membranes for cell culture in combination with other materials^21^. The lung can be modelled using air liquid interface on chip^22,23^. As PDMS chips are flexible, contractions can be simulated using over and under pressure to stretch the cells to improve the function^22,24^. Another key feature of organ on a chip technology, which does not require continuous flow, is the ability to position cells in 2D or 3D with micrometre precision. Many of these chips are operated without flow and are therefore not covered in this meta-analysis.

A very attractive development in terms of usability is to use fluidic hybrids where organ models are added to fluidic network as individual Transwell like inserts^25,26^. This fluidic architecture is compatible with many if not most technologies in tissue engineering and regenerative medicine^27,28^. Chips driven by gravity, so called pumpless chips, are also an interesting alternative for some applications because of the ease of use and the simplicity to scale the number of cultures^29–33^. Importantly, the flow can be made unidirectional^32,34^ to ensure that cells are exposed to the same flow direction all the time. There are possibilities to interconnect up to 14 organs together with pumpless fluidics^35,36^ or Transwell/fluid hybrids^25,26^ which would model a large part of the human physiology.

Culturing cells in microfluidics systems has been done for decades with increasing scientific output every year. With that large body of articles about cell culture on chip, it is possible to make a quantitative comparative study across the literature between perfused cultures in chips and corresponding static cultures. The pertinent question is: does the complexity and expense of *perfused* microfluidics cell culture systems pay off in terms of the functions of the resulting cell model?

## Methods

**Figure 2:**
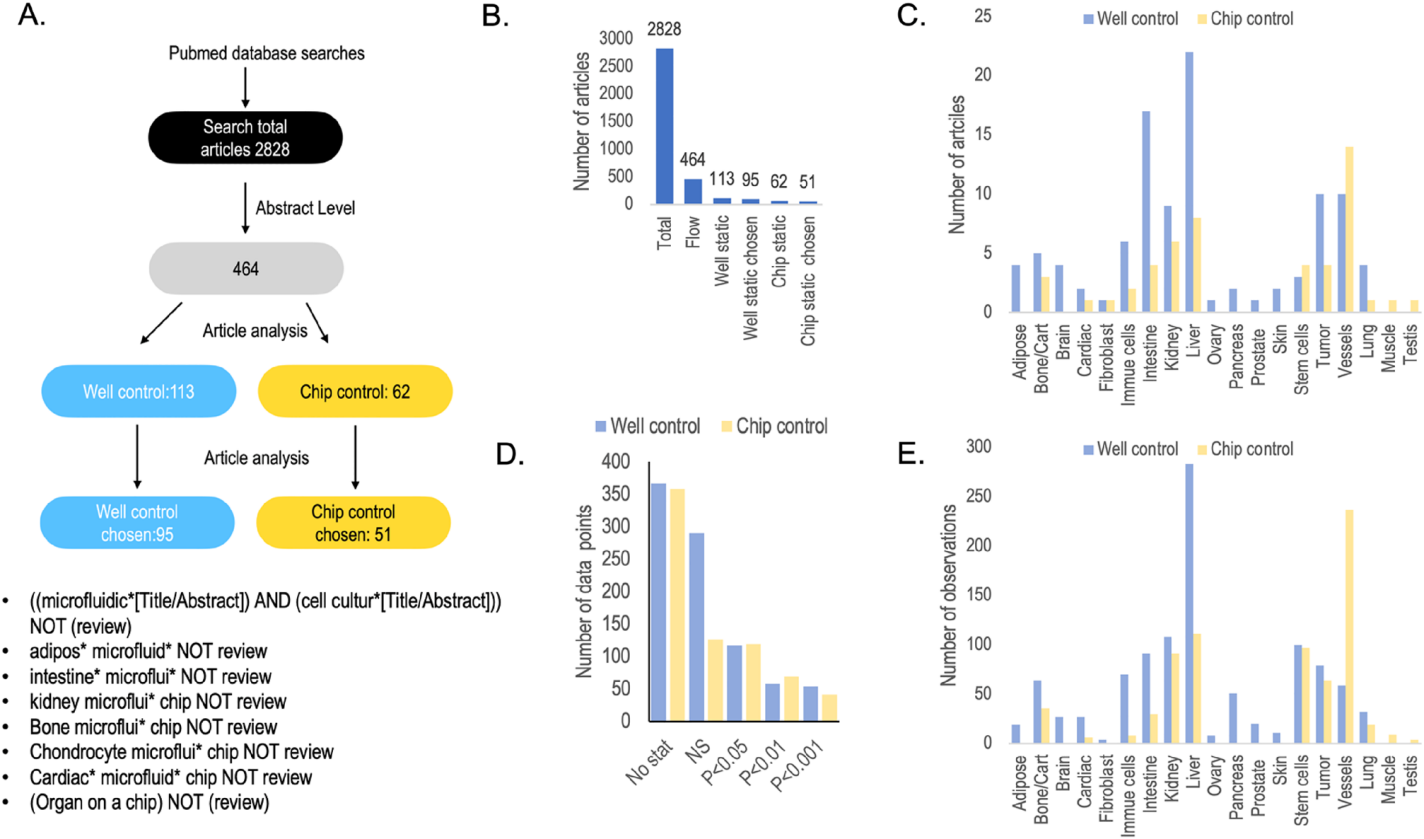
Summary of the study. (**A**) flow diagram of the selection of articles. (**B**) The number of scientific articles screened and selected. (**C**) The number of articles using the respective tissue. (**D**) The number of data points where the significance was tested and on which level. “NS”—tested but no significance “No stat”—no statistical test was performed. (**E**) The number of biomarker ratios per tissue that could be calculated from the data in the literature.

PubMed central (https://pubmed.ncbi.nlm.nih.gov/) was searched with the search strings presented in and in Fig. 2A and Supplementary Table S1. The inclusion criteria were articles published 2021 and earlier where the authors have used perfusion through a chip or recirculation within a chip for cell culture, have compared dynamic incubation to a proper static control, and have collected quantitative data. The static control was either a well, plate, Transwells or the chip itself. Graphs were copied into Keynote (Apple Inc.) and a line was used to read the graph on the Y-axis. If the axis scale was difficult to use directly, pixel counting of distances in the respective graphs was used. A benchmark between the pixel counting method and the quicker direct read showed a deviation of less than 5%. The data were transferred into Microsoft Excel for analysis. Supplementary Tables S1–S4 contain all the extracted data. The data were analysed as described in the result section using Excel functions such as t-test (paired, two tails), median and average. Time series were analysed by forming a ratio between flow and static culture at each time point. So, one time series resulted in many ratios. Every second point was used especially for time series with > 5 time points. The same strategy was used for dilution series. The motivation for that approach was a balance of over sampling from one article and under sampling by just including for instance end points or maximum difference. It should be noted that in many cases, the cultures were treated in some way for the cell to react and therefore the ratio did not only reflect “normal” cell culture vs corresponding flow-based culture but also the perturbed cultures. Each point in such a treatment matrix (control and treatment X, Y and Z) was calculated as a ratio between perfusion and static culture for the same exposures to see if perfusion enhanced the treatment or not. Hence, the ratio shows the deviation in response in static and dynamic cultures respectively but otherwise treated in the same way.

## Results and discussion

### Description of the collected data

A literature review was made with the aim to extract quantitative data where microfluidic cultures under flow were compared to corresponding batch cultures. 2828 articles were found (Fig. 2A,B, Supplementary Table S1) using first a wide unbiased search criteria and then a second search focusing on some tissues (adipocytes, vessels, intestine, liver, kidney, cardiac, bone, chondrocytes). A third search was performed where the more modern term “organ on a chip” keyword was used. This search terms overlapped greatly with the keyword “micro physiological system”. The selection for relevant articles was done on the title and abstract level and out of the 2828 articles, 464 articles describe usage of *perfusion* in cell culture. Many of the articles that were *not* included in the primary selection described droplet fluidics, integrated sensors, or static microfluidics cells culture. These articles had no data on perfusion, lacked cell culture data or lacked a suitable static control and were therefore excluded. After reading the 464 selected articles, 175 articles were shown to have the correct controls in term of static culture in “wells” (cell culture plate, dishes, wells, Transwells) or the “chip” itself. Of these, 95^15,20–22,37–126^ were selected as they included quantitative data from wells as controls and did not include physical cues like stretching the cell layer or electrodes for electrical stimulations. An additional 51 usable articles were found where the authors have used chip as static controls to the corresponding perfused chips^44,52,59,70,81,115,119,127–170^. The “well” and “chip” groups were compared to evaluate the impact of the chip material and confined cell culture spaces. Static incubation on chip will measure effects of very small medium height above the cells. Often, the chambers in the chips are only 100–200 µm high compared to wells where the medium above the cells is 1–2 mm high. Chips might also have low O_2_/CO_2_ exchange rates compared to open wells and perfusion which would affect both the pH and the respiration. The cell culture conditions that were included in the quantitative analyses are annotated with a tick mark in Fig. 1. The only feature that is not easily achieved in static cultures is a continuous stable gradient of factors that are used to investigate for instance migration and differentiations. It is noteworthy that so many articles describing perfusion cell cultures did not contain the correct static controls and therefore did not provide data to the study. My own reasons for not using static controls were either that the engineering the platform or chip was the focus^16,171^ or that the biology required perfusion cultures as the correct control^172^. Many authors seem to have made similar decisions.

Cells from different tissues are not covered equally much in the literature (Fig. 2C, blue bars). The shear sensitive barrier tissues such as intestine, lung, kidney and vessels are overrepresented. The metabolic active liver is also well represented in the literature. The number of articles with chips as static control follows the same bias in tissue usage (Fig. 2C, yellow bars). The number of extracted biomarkers data points per tissue correlates with the number of articles per tissue (Fig. 2E). However, frequency plots show a large spread in the number of biomarker data points gathered in the respective article (Supplementary Fig. 1). The largest fraction of articles had only up to five biomarker ratios described.

The statistical significance of the ratios in the respective article were also recorded (Fig. 2D). About half of the ratios between flow and the corresponding static culture in the literature were tested for statistical significance. Few showed high statistical significance in the differences between static and flow cultures for a biomarker and relatively many showed no statistical significance in biomarker response. This indicates that the data supporting that flow in chips is providing a benefit to the cell culture, is statistically weak and needs further investigations.

### Effects of perfusion

**Figure 3:**
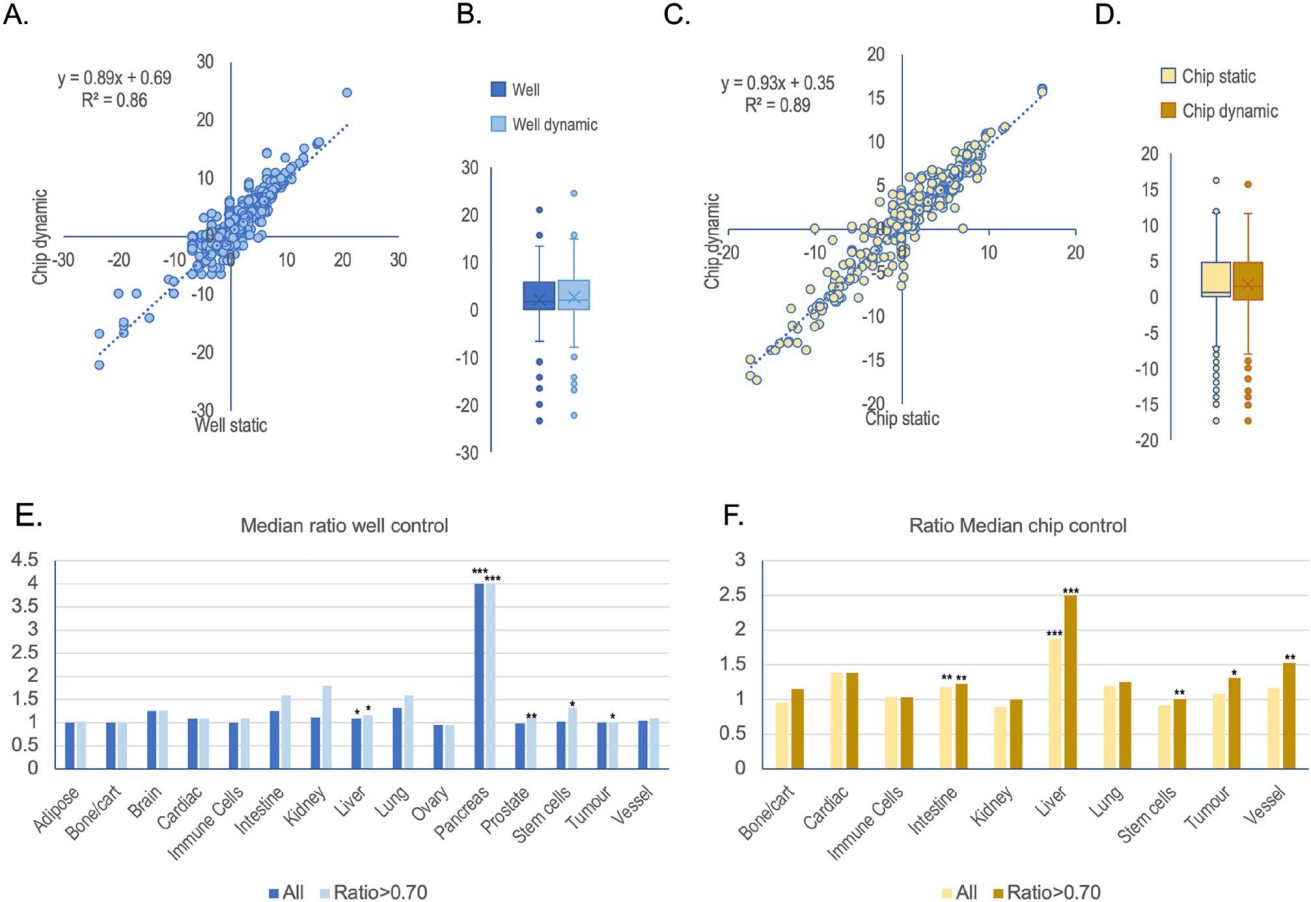
(**A**) Scatter plot of raw extracted data from well static controls and chips under perfusion or recirculation (Dynamic cultures). The axes show log2 transformed data. (**B**) Corresponding boxplot of the data in (**A**). (**C**) Scatter plot of raw extracted data from chip static controls and perfused chips. (**D**) Corresponding boxplot of the data in (**C**). The median biomarker ratio value per tissue for the wells (**E**) and chip static controls (**F**) respectively on a linear scale. “All” means all data and “> 0.7” means raw data that forms a ratio above 0.7 when biomarker activity obtained in a chip under flow is divided with the respective static control. *P < 0.05, **P < 0.01, ***P < 0.001 using t-test on raw data on from dynamic and static culture respectively.

Extracted data were plotted in scatter plots to identify larger trends in the effects of perfusion. There is a linear relationship between the data from the dynamic chips and the corresponding static controls (Fig. 3A,C). The scattering suggests that flow had an impact on the activity of some biomarkers but not all. The box quartile plots (Fig. 3B,D) show that only the median statistic (dash) differed between flow and static incubations respectively. Hence, flow incubations might give a better performance compared to static cultures, but increased performance is likely associated with few biomarkers or cell types as indicated by the outliers and whiskers.

The ratios between the microfluidic value and the corresponding static culture were calculated (see Supplementary Tables S2 and S3) to assess effects of dynamic incubation in chips. Furthermore, the datasets were binned into different cell types to identify if flow was beneficial to cells from one tissue but not others. The *median* ratios are all between 1 and 2 on the linear scale using wells or chips as static controls suggesting only a minor positive effect of dynamic incubations (Fig. 3E,F). The exception is pancreas, but this observation is based on only one article.

Since a chip under flow can have positive effect resulting in a fraction lower than 1 (for instance permeability over the intestine wall where the improved barrier function results in lower permeability) these lower ratios would even out the high ratios in the analysis and hence the net effect would be close to a ratio of 1 (as suggested in Fig. 3E,F). Therefore, the biomarkers giving rise to lower numerical values by flow were removed using a < 0.7 ratio cut-off. These cut-offs were used to allow for natural variations around 1 but also remove data of biomarkers that results in down regulations even if the down regulation is a good feature. Removing the ratio < 0.70 results in a higher median ratio value for some tissue (intestine, lung, vessel, liver, and stem cells) as compared to the whole data set (Fig. 3E,F, light coloured bars. Most tissues have more than 20 observations (Fig. 2E), so it was valid to calculate a students paired t-tests between *raw* data from the chip with flow and the corresponding static controls for the respective tissue. Statistically significant effects (P < 0.05) across the literature are observed in cells from tissues that were exposed to shear (intestine, vessel) and tissues with known high metabolic rates (liver) in vivo (Fig. 3E,F).

**Figure 4:**
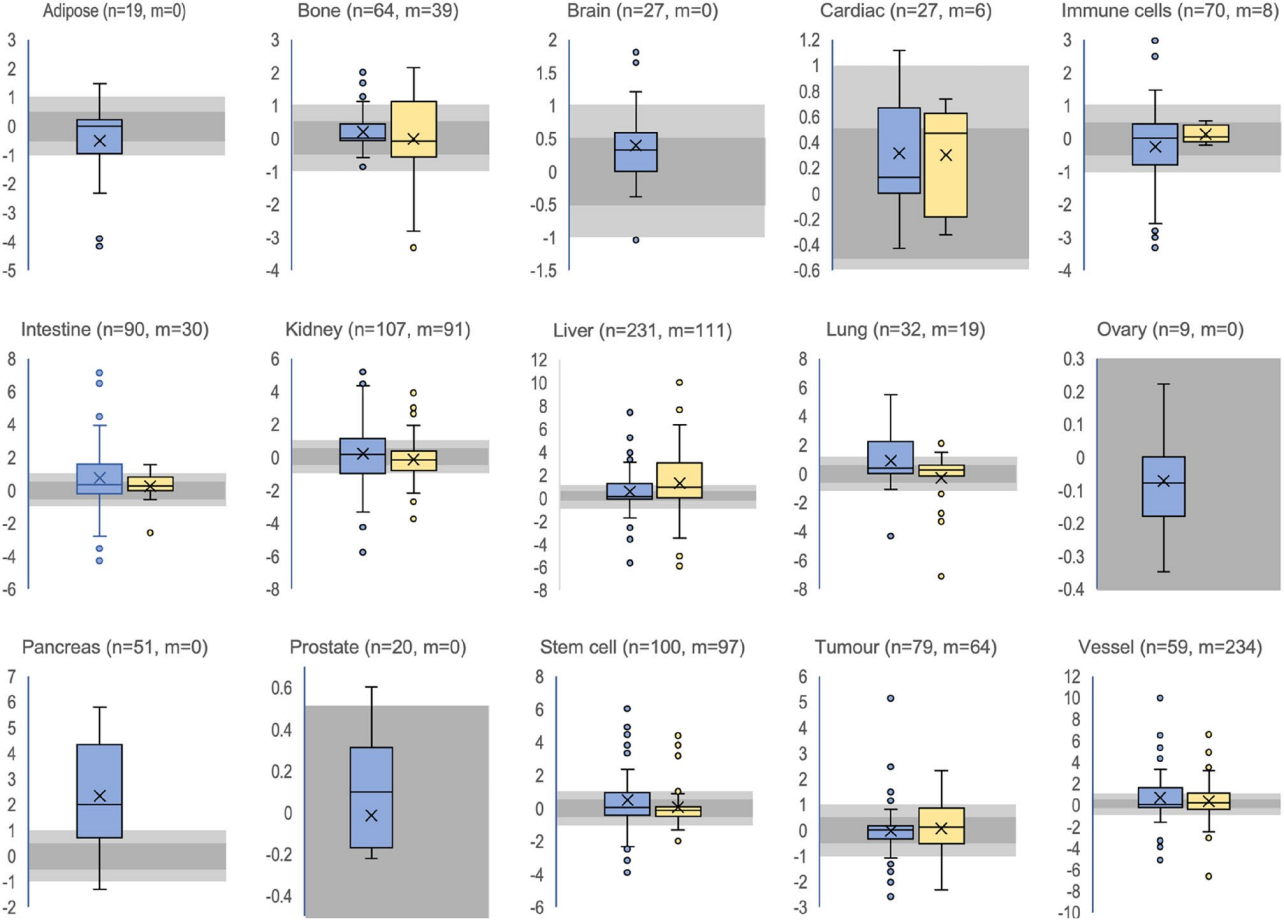
Boxplots of biomarker regulation (log2 ratio) in different tissues by flow. Blue shows the data with wells as static controls and yellow shows data with chips as static controls. “n” indicates the number of observations for wells as static controls and “m” indicated the number of observations for chips as static control for the respective biomarker. “n” and “m” are also reported in Fig. 2E that gives a better overview. Lightly grey shaded area around 0 indicate two-fold up and down regulation respectively (log2(2) = 1 and log2(0.5) = − 1). Darker grey shaded area around of indicate 0.7–1.4-fold regulation (log2(~ 0.7) = − 0.5 to log2(~ 1.4) = 0.5) respectively.

The data was split into wells as static controls (Blue, Fig. 4) and chips as static controls (Yellow, Fig. 4) and plotted as log2 transformed boxplots. This gave an insight into the spread of the ratio and therefore indicate the degree of regulation of biomarkers per tissue. Many tissues including those exposed to shear in the body seem to have most of the data points closely scattered around 0 (Log2(1)) suggesting that many of the investigated biomarkers in the respective tissue are not regulated by flow. Many tissues have whiskers above 1 and below − 1 respectively suggesting that several biomarkers are somewhat regulated by flow. Furthermore, many tissues have outliers (dots) suggesting that there are some biomarkers that are strongly regulated by flow. It is however not consistent as some regulation seem to happen with wells as control but not chips as control and vice versa. The reason is likely that too few papers are contributing to a specific tissue (Fig. 2C) or that different cell types or other experimental conditions might dominate the results instead of flow (see also below).

### Distributions of biomarker ratio

**Figure 5:**
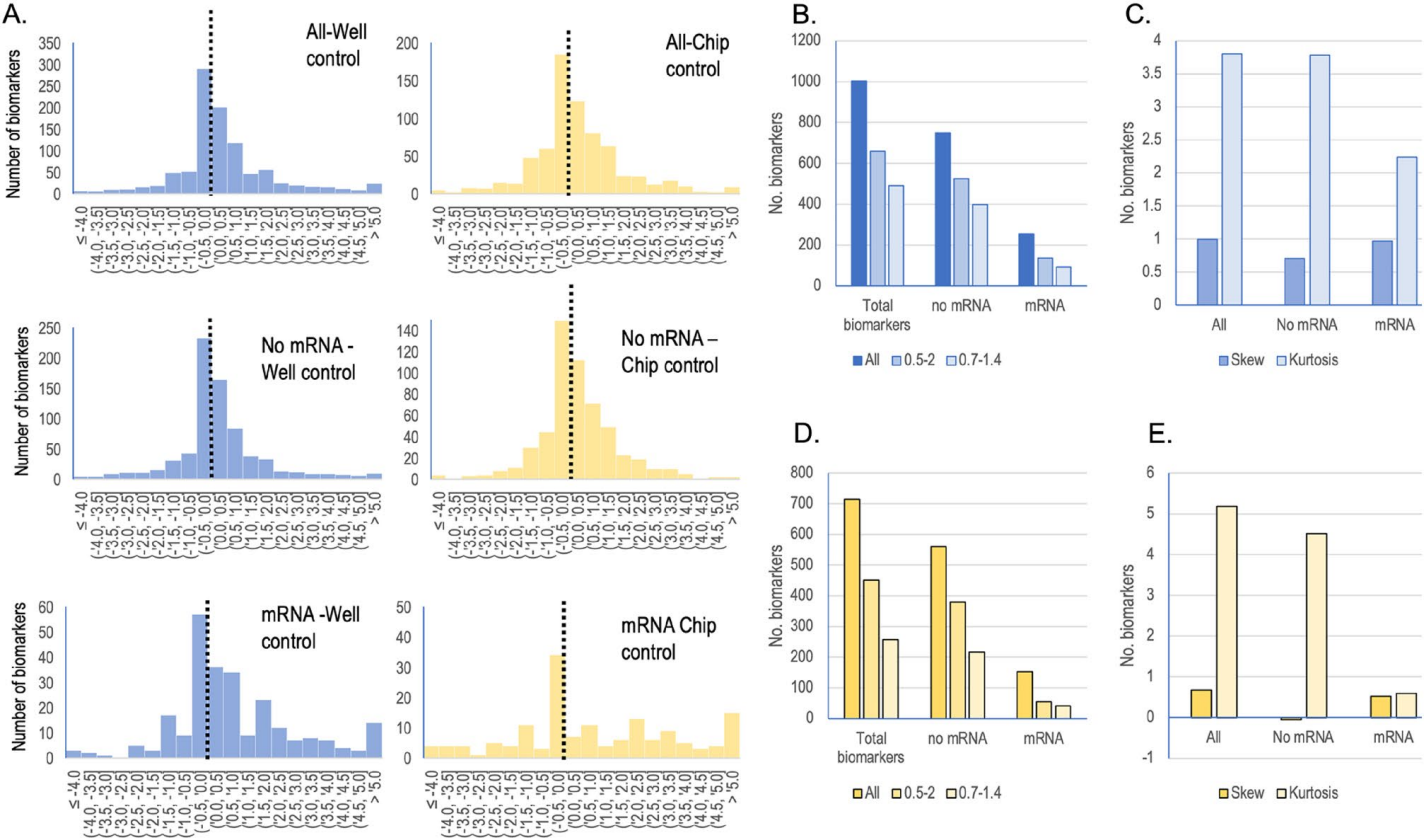
Analysis of biomarker distributions of Log2(ratio) data. (**A**) Histograms showing the distribution of ratio for biomarkers. “All” includes all the biomarker data from wells (blue) and chips (yellow) as static control, respectively. “No mRNA” is all the biomarkers but excluding the mRNA data. “mRNA” only includes mRNA data. (**B**) The number of observations within a given ratio interval on the linear scale. Strong blue includes all ratios, medium blue includes ratio interval log2(~ 0.7) = − 0.5 to log2(~ 1.4) = 0.5 and light blue includes the ratio interval log2(0.5) = − 1 to log2(2) = 1. (**C**) Calculated skewness and kurtosis on the respective distributions for wells as control using the respective data subset described in (**A**). (**D**) The number of observations in each ratio interval for chip control. See (**B**) for interval explanations. (**E**) Calculated skewness and kurtosis on the respective distributions for chips as controls.

The distributions of ratios were plotted, and skewness and kurtosis were calculated (Fig. 5) to get a detailed picture of impact of flow and possible bias. The most common ratio interval is from − 0.5 to 0 (log2 scale, Fig. 5A) but the distribution mostly followed a normal distribution. The chip as static control has similar distribution as the wells as control. 62% of the data points fall within a ratio range of 0.5–2 (linear scale) for the well controls. Corresponding value is 58% for chip controls. 38% and 32% respectively of the data points are within the range of ~ 0.70–1.4 (Fig. 5B,D). It is a similar pattern for the “mRNA” and “No-mRNA” subsets for both the wells as control and the chip as control. The skewness was calculated and shows the “All” data set is skewed towards + 1 (Fig. 5C) suggesting that overall, the biomarker chosen typically reacted positively to flow. However, mRNA data has also a skewness of + 1 while the “No mRNA” dataset has skewness of less than 1 (Fig. 5C). The chip as control shows similar pattern to wells as controls (Fig. 5E) but skewness is even more pronounced. Therefore, it is the large set of mRNA biomarkers that contribute largely to the positive skewness of the data sets (Fig. 5C,E) and may explain the improvement induced by flow in some tissues (Figs. 3E,F and 4). The kurtosis analysis suggests that all log2 transformed distributions are close to a normal distribution (value of 3, Fig. 5C,E). However, the kurtosis is larger than 3 so there is a tendency that more biomarkers are not regulated than would be expected from a normal distribution. In conclusion, this suggests that many biomarkers are unregulated by flow and just a few shows stronger regulations.

**Figure 6:**
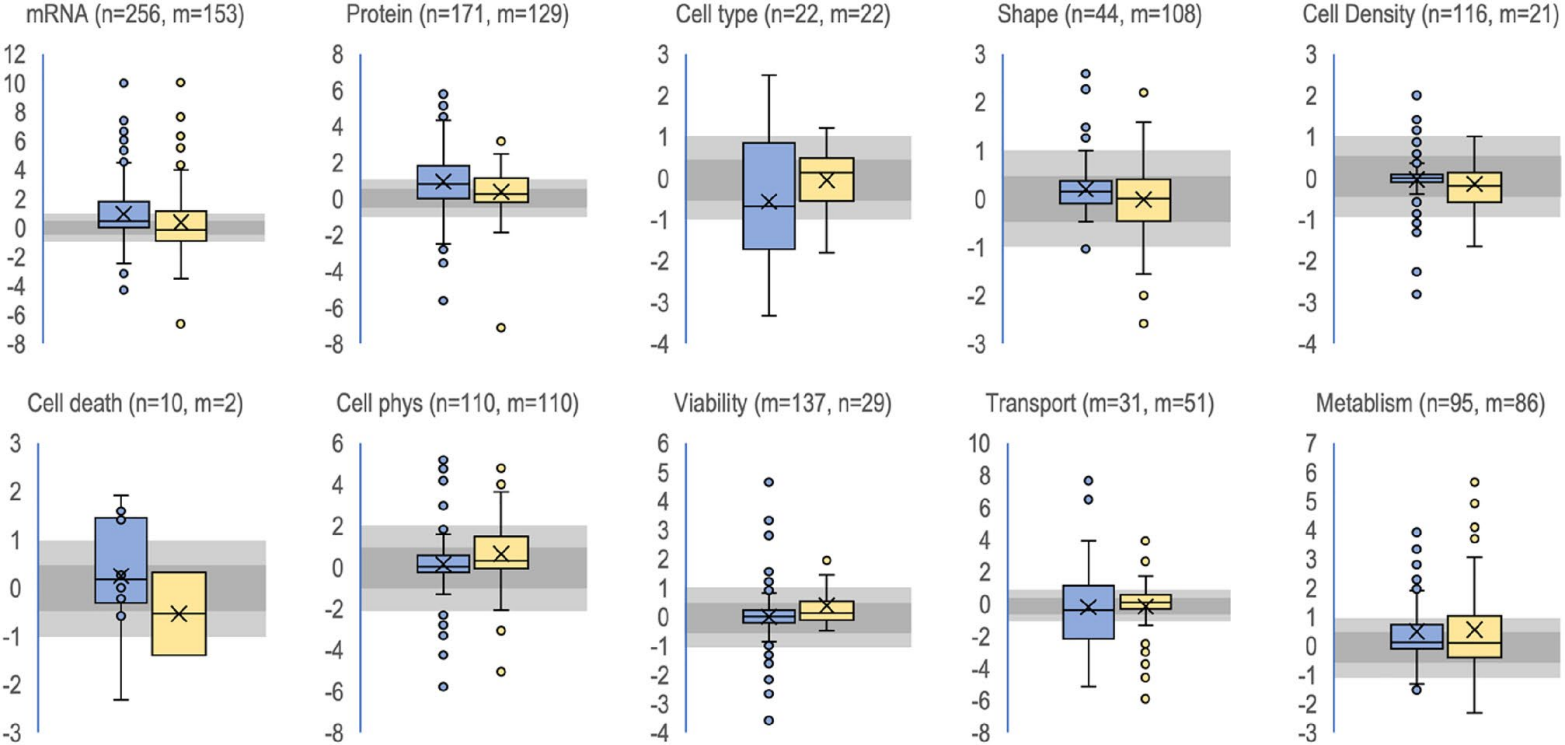
Regulation of biomarker classes by flow. The data is log2 transformed. Blue shows wells as static controls and yellow shows chips as static controls. “Cell physiology” include a collection of higher-level cell functions not covered by the other functions in the figure. “n” indicates the number of observations for wells as static controls and “m” indicated the number of observations for chips as static control for the respective biomarker. Lightly grey shaded area around 0 indicate two-fold up and down regulation respectively (log2(2) = 1 and log2(0.5) = − 1). Darker grey shaded area around of indicate 0.7–1.4-fold regulation (log2(~ 0.7) = − 0.5 to log2(~ 1.4) = 0.5) respectively.

Boxplots of the regulation of individual biomarker classes show that some biomarkers classes responded to flow as indicated by large spread of box and whiskers around 0 (log2(1)) (Fig. 6). Examples are cell metabolism, mRNA, protein, cell physiology parameters, and transport. Others had low spread around 0 suggesting no or little response to flow. Examples are viability, cell proliferation, and shape. All biomarker classes have some outliers suggesting that there are responses to flow in unique cases. The chips as static controls and the wells as static controls have mostly the same pattern indicating no large bias of one control over the other.

One explanation for that biomarker class generally do not show large regulations would be that specific markers of key functions have not been measured or that key regulated markers are few as compared to the measured non-regulated markers. This would make the data set biased towards non-regulated biomarkers and thus would collectively dominate the statistics. However, Fig. 5 suggests that > 40% of the biomarkers are regulated by flow to some extent which may indicate a positive selection bias towards regulated biomarkers but that is not affecting the median ratio value much (Figs. 2E,F and 4). Hence the data supports a model where cells under flow are in most cases the same as in static controls but differs on some key biomarkers.

It is, however, possible that cells under flow are in an adaption process from static cultures and it is expected that adaption should be observed firstly on the mRNA level and then on the cell physiology level as the latter demands many gene transcriptional changes. The observed flat mRNA distributions (Fig. 5) support this notion. If that is the case, the flow might have larger impact than currently being measured, but it also means that cells must be maintained under dynamic regime before a flow experiment. There is no article in the data set that subculture cells under dynamic conditions prior to the chip experiment.

### Identifying specific biomarkers that are regulated by flow

**Figure 7:**
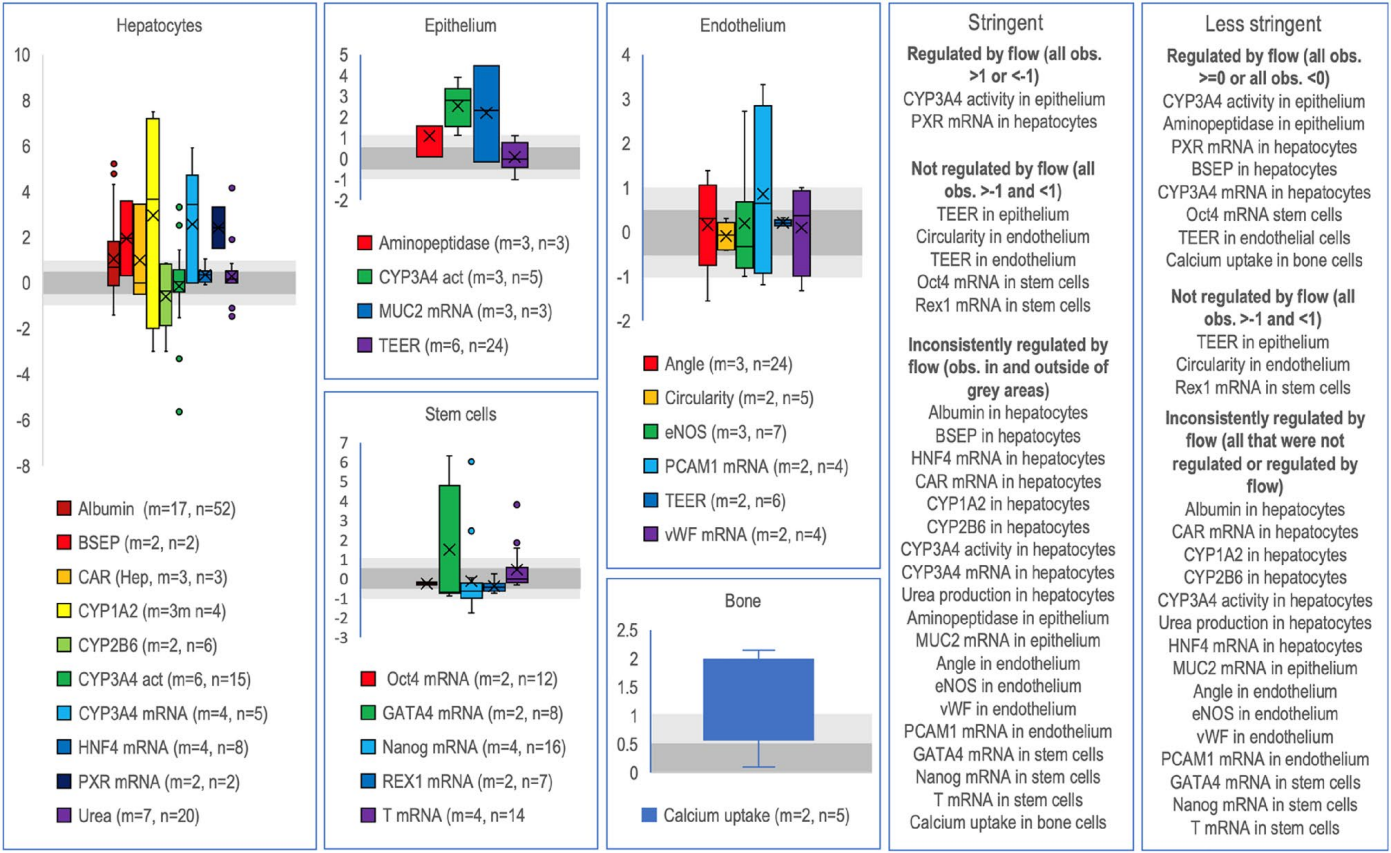
Log2 ratio boxplots for each specific biomarker and cell type. The data from chips and wells as controls were pooled for the respective biomarker. “m” indicates the number of publications and “n” indicates the total number of observations in the publications for the respective biomarker. Lightly grey shaded area around 0 indicate two-fold up and down regulation respectively (log2(2) = 1 and log2(0.5) = − 1). Darker grey shaded area around of indicate 0.7–1.4-fold regulation (log2(~ 0.7) = − 0.5 to log2(~ 1.4) = 0.5). The specific biomarkers were categorised into three classes based on the deviation from two-fold regulations (stringent) or being above 0 (less stringent). If a biomarker had observations both inside and outside the light grey area, they were classified as inconsistently regulated.

Next, biomarkers that were measured in at least two independent articles and on the same or similar cell types were identified. Here the data from the chip and wells were pooled to identify biomarker that where consistently regulated by flow (Fig. 7, Supplementary Table S3). 26 biomarkers were analysed in at least two different articles for a given cell type. Only the CYP3A4 activity in Caco2 cells and PXR mRNA in hepatocytes were *consistently* upregulated by flow across the literature (Fig. 7) using a stringent cut-off of more or less than twofold regulation. A less stringent classification suggest that eight biomarkers were consistently regulated by flow, and some are expected such as CYP3A4 mRNA expression in hepatocytes. This gene is known to be induced by some chemicals^173^ and the effective concentration of chemicals will be higher with mixing or flow. Several biomarkers were *consistently unaffected* by flow (Fig. 7) such as TEER in both endothelium and epithelium. It is noteworthy that TEER in Caco2 cells is not affected by flow suggesting that some parameters are affected (CYP3A4 activity) and others are not (TEER) in the same cell line. The reason is unclear, but one explanation is that the CYP3A4 activity is limited by the mass transfer of the substrate under static conditions. The flow will increase the mass transfer rate and thereby explain the apparent increase in activity. Many biomarkers were *inconsistently* regulated by flow both using a stringent or less stringent classification. Analysing the article-by-article distribution per biomarker and cell type (Supplementary Fig. 3) suggested that distributions in 52 of totally 95 articles in this subset did not overlap for a given biomarker. This suggest a large lab-to-lab variation. One is albumin secretion in hepatocytes where the same cell type reacts differently as indicated by the large spread (Fig. 7). However, the between article-to-article variation of albumin secretion is also large (Supplementary Fig. 3). Analysing the article where albumin was induced suggests that the static chip control performed very poorly in many parameters^169^. This suggests that poor performance of the static control perhaps in combination with a true upregulation of the activity gives collectively the appeared better performance of flow. Hence this chip architecture, perhaps due to the very small volumes used, was very dependent on flow to provide suitable cell culture conditions. I another paper^85^, the flow seems to affect CYP3A4 activity in hepatocytes negatively (Fig. 7, Supplementary Fig. 3). The reason is likely that the cells in the static controls were grown as spheroids that apparently had higher activity than cell on the chip under flow. There are other possible reasons for inconsistent regulations such as cell to cell variability, different flow velocities of the medium, chip materials and architecture that might differ between the different articles.

### Impact of flow regimes and 2D and 3D cultures

**Figure 8:**
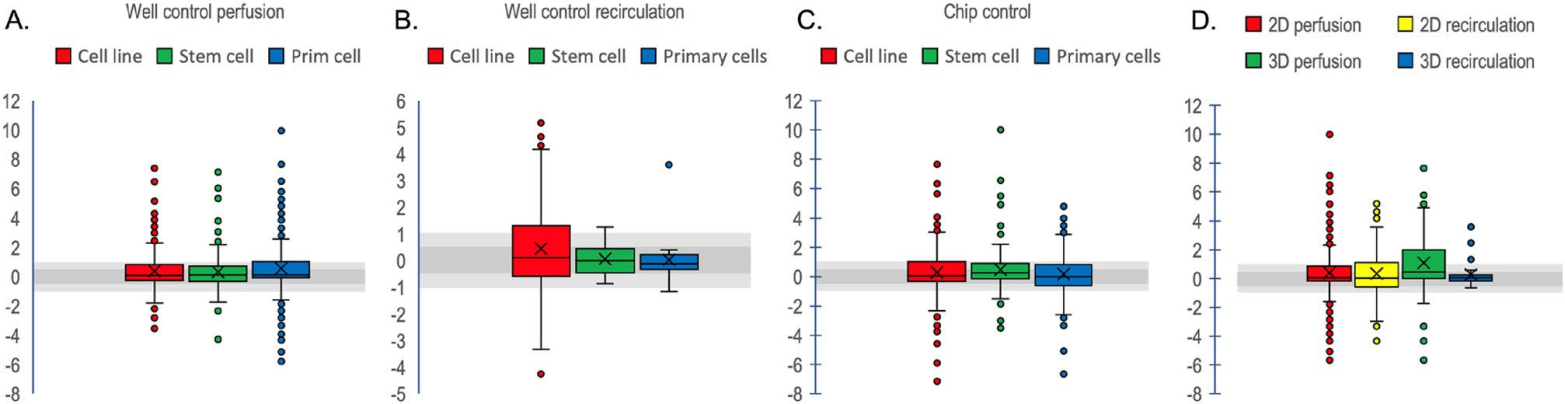
Box plots of ratio of different cell classes under perfusion where the medium passes the chips once with wells as control (**A**), is recirculated on chip with wells as control (**B**) or perfused using a chip as control (**C**). (**D**) Comparison of perfusion, recirculation on 2D and 3D cultures respectively using wells as controls.

Cell classes and perfusion classes were next analysed to see if there was any pattern between for instance rapidly dividing cells lines and the perfusion method. Therefore, the data was split into primary cells, cell lines (immortalised) and stem cells, respectively. These where split into chip static controls, and perfusion or recirculation using wells as controls. All cell classes and perfusion methods have a narrow ratio distribution close to 1 indicating that the perfusion type is mostly not affecting function (Fig. 8A–C). This suggest that in all conditions, including all static cultures even in chips, cells were normally fed sufficiently. The whiskers and outliers indicate as usual that specific biomarkers are sensitive to flow. However, an ANOVA test of 3D vs 2D growth in perfusion and in recirculation respectively using data from wells as controls indicated a statistical difference. A t-test for each pair suggested that perfusion of 3D cultures had higher activities of the cells (green bar Fig. 8D). Except for this case, it seems that cells are nearly indifferent to the perfusion or feeding strategy. This means that we can expect that cells mostly behave the same in fluidics as compared to static cultures and only some biomarkers are affected by flow. Exceptions are when flow breaks paracrine signalling which may have large consequence for cell function^172^.

The magnitude of flow rate and resulting shear was furthermore investigated (Supplementary Fig. 4)^115,130,145,151,164^. It appears that some biomarkers reacted strongly to increased shear (or feeding) induced by higher flow rates while other biomarkers barely reacted. It should be noted that only 5 of the included 146 articles analysed the magnitude of shear or feeding rate on cellular functions suggesting that further optimisation should be done.

The most medium restricted cell culture condition is the static chip control due to the limited amount of medium in the system. The chip static controls are likely successful because the most common chip material is the gas permeable PDMS which would ensure sufficient O_2_ exchange even during the culture in shallow channels. Calculating the glucose usage during 24 h from 2D cell culture data^174^, suggests that 100–200 µm medium height above the cells per day is used up which is often the height of the medium column above the cells in a chip. In most cases, the medium was exchanged every day in chip controls suggesting that cells are fed sufficiently even in static chips. Hence medium is not limiting many of the functions of cells in chips in static cultures. This is expected as the cell medium has been optimised for static incubations of 2D cultures and is therefore relatively rich. The metabolic rate of other substrates such as those metabolised by CYP enzymes (Fig. 7) might, by contrast, be diffusion limited, which might explain higher activities under perfusion of substrates for CYP3A4. Diffusion limitations might also explain the advantage of perfusion on 3D cultures as static culture or recirculation might create a diffusion limited feeding due to the many cell layers in for instance spheroids.

If diffusion limitation is the reason for improved activities, the wells could be agitated using minor investments like the setup used for drug transport studies which essentially is an orbital shaker or a rocker unit in an incubator. It is striking that of the 95 articles with well controls, 94 used static batch cultures as controls and 1 used agitate wells as controls. Agitation using for instance an orbital shaker would nearly remove any vertical and time gradients of secreted factors, waste, and nutrients.

### Considerations for transition to microfluidics based systems

Moving into the field of microfluidics-based cell culture is costly, has a steep learning curve and hampers the throughput so before taking the step, there are many considerations to do based on this meta-analysis. Firstly, do not expect the cells to be widely different from the corresponding batch cultures at least not when grown under flow for relatively short periods. This is despite the exposure of shear, improved mass transfer, and exposure to other cell growth substrate such as PDMS. This suggests that most of the prior knowledge collected from static cultures of the respective cell models can be applied. Secondly, ensure that the biomarker in question is reacting to flow or shear in a way that improves the desired function sufficiently much. The literature is far from clear (see Figs. 2, 3, 4 and 7) in this respect nor is it clear that necessary optimisations regarding the magnitude of flow rate applied has been done (Supplementary Fig. 4). In that context, the observed variation in responses in the respective article (Fig. 7 and Supplementary Fig. 3) to flow might be lab to lab variation and different sources of cells as well as the differences in the chip design or how it was operated. Thirdly, medium optimisations are very rarely done in combination with flow and hence there is a risk of over feeding rare compounds such as differentiation factors^172^ which may result in waste or even decreased cellular function. Diffusion limited situations such as 3D cultures and possibly CYP3A4 metabolic activity (Figs. 7 and 8) may react positively to perfusion where constant new medium supply is provided. However, perfusion where the medium passes only once is very costly. At 10 µl/min, a chip uses 14.4 mL in 24 h but lower flow rates such as 1 µl/min might be sufficient, or the medium can be recirculated without large effects excepts at very large cell densities (Fig. 8). Even in recirculation setups, the dead volume are often larger than in static cultures due to large dead volumes. Another drawback of high medium usage is that analytes are diluted. While OOC claims physiological relevance, the medium to cell ratio is not nearly the ratio between blood/interstitial fluid to cell ratio in the body which may affect pharmacometrics but also the concentration of secreted autocrine and paracrine factors which will be too diluted to activate cells. Fourthly, evaluate the throughput needed. Pumps and chips are expensive. Investigate if there are other commercial cell culture devices available including gravity-based systems such as those from Mimetas, inSphero and Akita or even mixing plates through agitation or similar solutions, that allows for higher throughput and provide some if not most of the benefits of pump-based microfluidics. Fifthly, does the coculture and positioning of the respective cell type need micro-meter precision to simulate a tissue? This is one of the stronger points of microfluidics but as illustrate here (Fig. 8) does not necessary require perfusion to provide excellent cell models (not reviewed here but warrants a similar meta-analysis). As mentioned above, cruder cell positioning is also possible in plates and Transwells^7–9^. Finally, is it desirable to connect different organs or tissue models together and is the medium routing speed and volume between tissue models important? The different OOC coculture models are challenging and is highly driven by pharmacometrics^56,58,89,109,138^ rather than other types of organ-to-organ communication that does not necessary involve the liver for breakdown of compounds. The pharmacodynamics is often fast such as hours or less while endo- and paracrine signalling could be far slower and could likely be supported by slower means of transport than flow such as diffusion over short distances.

## Conclusions

In conclusion, the evidence that chips need to be perfused is weak due to lack of statistics in the respective articles (Fig. 2D). Although, the cross-literature analysis performed here suggest some possible gains by flow (Figs. 3, 4, 5) but in all cases except a few (Fig. 7), these gains have not been verified by another laboratory. Cells from tissues reacting to shear or have high metabolic rate in vivo is overrepresented in the literature (Fig. 2). These tissues are also those that show some effects of flow (Figs. 3, 4, 5). Factors that likely are affecting gains are the cell types used (Fig. 4) and biomarker investigated (Fig. 7). It is more difficult to link gains to method of incubation except for 3D cultures that work slightly better under perfusion (Fig. 8). Is the complex chips and perfusion needed? The analysis suggest that it is, but only in some specific cases using some specific cell types and biomarker combinations.

## Supplementary Information

Supplementary Information.

Supplementary Table S1.

Supplementary Table S2.

Supplementary Table S3.

Supplementary Table S4.

## Data Availability

All data generated or analysed during this study are included in this published article and its supplementary information files.

## References

[1] Ingber DE (2020). Is it time for reviewer 3 to request human organ chip experiments instead of animal validation studies?. Adv. Sci..

[2] Bhatia SN, Ingber DE (2014). Microfluidic organs-on-chips. Nat. Biotechnol..

[3] Skardal A (2017). Multi-tissue interactions in an integrated three-tissue organ-on-a-chip platform. Sci. Rep..

[4] McGonigle P, Ruggeri B (2014). Animal models of human disease: Challenges in enabling translation. Biochem. Pharmacol..

[5] Mak IW, Evaniew N, Ghert M (2014). Review Article Lost in translation: Animal models and clinical trials in cancer treatment. Am. J. Transl. Res..

[6] Hatherell K, Couraud PO, Romero IA, Weksler B, Pilkington GJ (2011). Development of a three-dimensional, all-human in vitro model of the blood-brain barrier using mono-, co-, and tri-cultivation Transwell models. J. Neurosci. Methods.

[7] Rodriguez-Crespo D (2014). Triple-layered mixed co-culture model of RPE cells with neuroretina for evaluating the neuroprotective effects of adipose-MSCs. Cell Tissue Res..

[8] Antunes F, Andrade F, Araújo F, Ferreira D, Sarmento B (2013). Establishment of a triple co-culture in vitro cell models to study intestinal absorption of peptide drugs. Eur. J. Pharm. Biopharm..

[9] Ito R (2019). A human immortalized cell-based blood-brain barrier triculture model: Development and characterization as a promising tool for drug-brain permeability studies. Mol. Pharm..

[10] Shafran, Y. *et al.* Co-culture hydrogel micro-chamber array-based plate for anti-tumor drug development at single-element resolution. *Toxicol. In Vitro*. **71** (2021). 10.1016/j.tiv.2020.105067 33301902

[11] Leth Jepsen, M. *et al.* Tissue Engineering: 3D Printed Stackable Titer Plate Inserts Supporting Three Interconnected Tissue Models for Drug Transport Studies. *Adv. Biosyst*. **4**, e1900289 (2020). 10.1002/adbi.201900289 32390341

[12] Driessen, R. *et al.* Computational characterization of the dish-in-a-dish, a high yield culture platform for endothelial shear stress studies on the orbital shaker. *Micromachines (Basel)*. **11** (2020). 10.3390/mi11060552 PMC7345652 32486105

[13] Dardik, A. *et al.* Differential effects of orbital and laminar shear stress on endothelial cells. *J. Vasc. Surg*. **41** (2005). 10.1016/j.jvs.2005.01.020 15886673

[14] Herland A (2016). Distinct contributions of astrocytes and pericytes to neuroinflammation identified in a 3D human blood-brain barrier on a chip. PLoS ONE.

[15] Parrish J, Lim KS, Baer K, Hooper GJ, Woodfield TBF (2018). A 96-well microplate bioreactor platform supporting individual dual perfusion and high-throughput assessment of simple or biofabricated 3D tissue models. Lab. Chip.

[16] Sabourin, D. *et al.* The MainSTREAM component platform: A holistic approach to microfluidic system design. *J. Lab. Autom*. **18** (2013). 10.1177/2211068212461445 23015520

[17] Obi S (2009). Fluid shear stress induces arterial differentiation of endothelial progenitor cells. J. Appl. Physiol..

[18] Olesen S-P, Claphamt D, Davies P (1988). Haemodynamic shear stress activates a K+ current in vascular endothelial cells. Nature.

[19] Levesque M, Nerem R (1985). The elongation and origenation of cultured endothelial cell sin response to shear stress. J. Biol. Eng..

[20] Kim HJ, Ingber DE (2013). Gut-on-a-Chip microenvironment induces human intestinal cells to undergo villus differentiation. Integr. Biol..

[21] Tan HY (2018). A multi-chamber microfluidic intestinal barrier model using CaCo-2 cells for drug transport studies. PLoS ONE.

[22] Huh D (2010). Reconstituting organ-level lung functions on a chip. Science.

[23] Bluhmki, T. *et al.* Development of a miniaturized 96-Transwell air–liquid interface human small airway epithelial model. *Sci. Rep*. **10**, 13022 (2020). 10.1038/s41598-020-69948-2 PMC7400554 32747751

[24] Huang, D. *et al.* Reversed-engineered human alveolar lung-on-a-chip model. *Proc Natl Acid. Sci. USA.* **118**, e2016146118 (2021). 10.1073/pnas.2016146118 PMC8126776 33941687

[25] Edington CD (2018). Interconnected microphysiological systems for quantitative biology and pharmacology studies. Sci. Rep..

[26] Maschmeyer I (2015). A four-organ-chip for interconnected long-term co-culture of human intestine, liver, skin and kidney equivalents. Lab. Chip.

[27] Zhu W (2016). 3D printing of functional biomaterials for tissue engineering. Curr. Opin. Biotechnol..

[28] Bakhshandeh B (2017). Tissue engineering; strategies, tissues, and biomaterials. Biotechnol. Genet. Eng. Rev..

[29] Chen, Z., He, S., Zilberberg, J. & Lee, W. Pumpless platform for high-throughput dynamic multicellular culture and chemosensitivity evaluation. in *Transactions of the Annual Meeting of the Society for Biomaterials and the Annual International Biomaterials Symposium* vol. 40 705 (Society for Biomaterials, 2019). 10.1039/c8lc00872h PMC6333476 30547180

[30] Lohasz, C., Frey, O., Bonanini, F., Renggli, K. & Hierlemann, A. Tubing-free microfluidic microtissue culture system featuring gradual, in vivo-like substance exposure profiles. *Front. Bioeng. Biotechnol*. **7** (2019). 10.3389/fbioe.2019.00072 PMC6454105 31001529

[31] Boos, J. A., Misun, P. M., Michlmayr, A., Hierlemann, A. & Frey, O. Microfluidic multitissue platform for advanced embryotoxicity testing in vitro. *Adv. Sci*. **6**, (2019). 10.1002/advs.201900294 PMC6662399 31380185

[32] Lee, D. W., Choi, N. & Sung, J. H. A microfluidic chip with gravity-induced unidirectional flow for perfusion cell culture. *Biotechnol. Prog*. **35** (2019). 10.1002/btpr.2701 30294886

[33] Langerak, N. *et al.* A theoretical and experimental study to optimize cell differentiation in a novel intestinal chip. *Front. Bioeng. Biotechnol*. **8** (2020). 10.3389/fbioe.2020.00763 PMC7393935 32793567

[34] Wang YI, Shuler ML (2018). UniChip enables long-term recirculating unidirectional perfusion with gravity-driven flow for microphysiological systems. Lab. Chip..

[35] Miller, P. G. & Shuler, M. L. Design and demonstration of a pumpless 14 compartment microphysiological system. *Biotechnol. Bioeng*. **113** (2016). 10.1002/bit.25989 27070809

[36] Esch MB, Ueno H, Applegate DR, Shuler ML (2016). Modular, pumpless body-on-a-chip platform for the co-culture of GI tract epithelium and 3D primary liver tissue. Lab. Chip.

[37] Bovard D (2018). A lung/liver-on-a-chip platform for acute and chronic toxicity studies. Lab. Chip.

[38] Ya S (2021). On-chip construction of liver lobules with self-assembled perfusable hepatic sinusoid networks. ACS Appl. Mater. Interfaces.

[39] Zhang J (2021). Construction of a high fidelity epidermis-on-a-chip for scalable: In vitro irritation evaluation. Lab. Chip.

[40] Miedel, M. T. *et al.* Modeling the effect of the metastatic microenvironment on phenotypes conferred by estrogen receptor mutations using a human liver microphysiological system. *Sci. Rep*. **9** (2019). 10.1038/s41598-019-44756-5 PMC6554298 31171849

[41] Chang SY (2017). Characterization of rat or human hepatocytes cultured in microphysiological systems (MPS) to identify hepatotoxicity. Toxicol. In Vitro.

[42] Patel, S. N. *et al*. Organoid microphysiological system preserves pancreatic islet function within 3D matrix. *Sci. Adv*. **7** (2021). 10.1126/sciadv.aba5515 PMC7880596 33579705

[43] Frost, T. S., Jiang, L., Lynch, R. M. & Zohar, Y. Permeability of epithelial/endothelial barriers in transwells and microfluidic bilayer devices. *Micromachines (Basel)*. **10** (2019). 10.3390/mi10080533 PMC6722679 31412604

[44] Lohasz, C. *et al.* Predicting metabolism-related drug–drug interactions using a microphysiological multitissue system. *Adv. Biosyst* **4**, (2020). 10.1002/adbi.202000079 33073544

[45] Qi, L. *et al.* Probing insulin sensitivity with metabolically competent human stem cell-derived white adipose tissue microphysiological systems. *Small*. **18** (2022). 10.1002/smll.202103157 PMC8776615 34761526

[46] Aziz, A. U. R. *et al.* A microfluidic device for culturing an encapsulated ovarian follicle. *Micromachines (Basel)*. **8** (2017). 10.3390/mi8110335 PMC6190016 30400524

[47] Liu, D. *et al.* Investigation of absorption, metabolism and toxicity of ginsenosides compound K based on human organ chips. *Int. J. Pharm*. **587** (2020). 10.1016/j.ijpharm.2020.119669 32702454

[48] Sances S (2018). Human iPSC-derived endothelial cells and microengineered organ-chip enhance neuronal development. Stem Cell Rep..

[49] Cherne, M. D. *et al.* A synthetic hydrogel, VitroGel® ORGANOID-3, improves immune cell-epithelial interactions in a tissue chip co-culture model of human gastric organoids and dendritic cells. *Front. Pharmacol*. **12** (2021). 10.3389/fphar.2021.707891 PMC8450338 34552484

[50] Maurer, M. *et al.* A three-dimensional immunocompetent intestine-on-chip model as in vitro platform for functional and microbial interaction studies. *Biomaterials*. **220** (2019). 10.1016/j.biomaterials.2019.119396 31398556

[51] Tao T (2019). Engineering human islet organoids from iPSCs using an organ-on-chip platform. Lab. Chip.

[52] Rauti, R. *et al.* Transforming a well into a chip: A modular 3D-printed microfluidic chip. *APL Bioeng*. **5**, (2021). 10.1063/5.0039366 PMC8084581 33948527

[53] Banaeiyan, A. A. *et al.* Design and fabrication of a scalable liver-lobule-on-a-chip microphysiological platform. *Biofabrication*. **9** (2017). 10.1088/1758-5090/9/1/015014 28155845

[54] Wang Y, Wang L, Guo Y, Zhu Y, Qin J (2018). Engineering stem cell-derived 3D brain organoids in a perfusable organ-on-a-chip system. RSC Adv..

[55] Shanti, A. *et al.* Multi-compartment 3D-cultured organ-on-a-chip: Towards a biomimetic lymph node for drug development. *Pharmaceutics*. **12** (2020). 10.3390/pharmaceutics12050464 PMC7284904 32438634

[56] Lee H (2017). A pumpless multi-organ-on-a-chip (MOC) combined with a pharmacokinetic-pharmacodynamic (PK-PD) model; A pumpless multi-organ-on-a-chip (MOC) combined with a pharmacokinetic-pharmacodynamic (PK-PD) model. Biotechnol. Bioeng..

[57] Xie, X. *et al.* Customizable microfluidic origami liver-on-a-chip (oLOC). *Adv. Mater. Technol*. **7** (2022). 10.1002/admt.202100677 PMC9231824 35754760

[58] Fetah K (2016). Pharmacokinetic profile that reduces nephrotoxicity of gentamicin in a perfused kidney-on-a-chip. Biofabrication.

[59] Choe A, Ha SK, Choi I, Choi N, Sung JH (2017). Microfluidic Gut-liver chip for reproducing the first pass metabolism. Biomed. Microdevices.

[60] Yen MH (2016). Efficient generation of hepatic cells from mesenchymal stromal cells by an innovative bio-microfluidic cell culture device. Stem Cell Res. Ther..

[61] Komeya M (2016). Long-term ex vivo maintenance of testis tissues producing fertile sperm in a microfluidic device. Sci. Rep..

[62] Hsieh CC, Huang SB, Wu PC, Shieh DB, Lee GB (2009). A microfluidic cell culture platform for real-time cellular imaging. Biomed. Microdevices..

[63] Zheng W (2016). An early-stage atherosclerosis research model based on microfluidics. Small.

[64] Huang SB, Wu MH, Wang SS, Lee GB (2011). Microfluidic cell culture chip with multiplexed medium delivery and efficient cell/scaffold loading mechanisms for high-throughput perfusion 3-dimensional cell culture-based assays. Biomed. Microdevices..

[65] Toh YC (2007). A novel 3D mammalian cell perfusion-culture system in microfluidic channels. Lab. Chip.

[66] Shi X, Zhou J, Zhao Y, Li L, Wu H (2013). Gradient-regulated hydrogel for interface tissue engineering: steering simultaneous osteo/chondrogenesis of stem cells on a chip. Adv. Healthc. Mater..

[67] Torisawa, Y. *et al.* Modeling hematopoiesis and responses to radiation countermeasures in a bone marrow-on-a-chip. **22**, 509–515 (2016). https://home.liebertpub.com/tec. Accessed 11 Oct 2022. 10.1089/ten.TEC.2015.0507 26993746

[68] Torisawa YS (2014). Bone marrow-on-a-chip replicates hematopoietic niche physiology in vitro. Nat. Methods..

[69] Snouber LC (2012). Analysis of transcriptomic and proteomic profiles demonstrates improved Madin-Darby canine kidney cell function in a renal microfluidic biochip. Biotechnol. Prog..

[70] Zhou M (2016). Development of a functional glomerulus at the organ level on a chip to mimic hypertensive nephropathy. Sci. Rep..

[71] Vriend J (2018). Screening of drug-transporter interactions in a 3D microfluidic renal proximal tubule on a chip. AAPS J..

[72] Theobald J (2019). In vitro metabolic activation of vitamin D3 by using a multi-compartment microfluidic liver-kidney organ on chip platform. Sci. Rep..

[73] Wang J (2019). A virus-induced kidney disease model based on organ-on-a-chip: Pathogenesis exploration of virus-related renal dysfunctions. Biomaterials.

[74] Yin L (2020). Efficient drug screening and nephrotoxicity assessment on co-culture microfluidic kidney chip. Sci. Rep..

[75] Kim HJ, Huh D, Hamilton G, Ingber DE (2012). Human gut-on-a-chip inhabited by microbial flora that experiences intestinal peristalsis-like motions and flow. Lab Chip.

[76] Marin TM (2019). Acetaminophen absorption and metabolism in an intestine/liver microphysiological system. Chem. Biol. Interact..

[77] Wang Y (2019). A 3D construct of the intestinal canal with wrinkle morphology on a centrifugation configuring microfluidic chip. Biofabrication.

[78] De Gregorio V (2020). Intestine-on-chip device increases ECM remodeling inducing faster epithelial cell differentiation. Biotechnol. Bioeng..

[79] Bein A (2021). Enteric coronavirus infection and treatment modeled with an immunocompetent human intestine-on-a-chip. Front. Pharmacol..

[80] Miller PG, Shuler ML (2016). Design and demonstration of a pumpless 14 compartment microphysiological system. Biotechnol. Bioeng..

[81] Lee DW, Choi N, Sung JH (2019). A microfluidic chip with gravity-induced unidirectional flow for perfusion cell culture. Biotechnol. Prog..

[82] Lohasz C, Rousset N, Renggli K, Hierlemann A, Frey O (2019). Scalable microfluidic platform for flexible configuration of and experiments with microtissue multiorgan models. SLAS Technol..

[83] Chen Z, He S, Zilberberg J, Lee W (2019). Pumpless platform for high-throughput dynamic multicellular culture and chemosensitivity evaluation. Lab. Chip.

[84] Lohasz, C., Frey, O., Bonanini, F., Renggli, K. & Hierlemann, A. Tubing-free microfluidic microtissue culture system featuring gradual, in vivo-like substance exposure profiles. *Front. Bioeng. Biotechnol*. 72 (2019). 10.3389/fbioe.2019.00072 PMC6454105 31001529

[85] Busche M (2020). HepaChip-MP—A twenty-four chamber microplate for a continuously perfused liver coculture model. Lab. Chip.

[86] Starokozhko V (2018). Differentiation of human-induced pluripotent stem cell under flow conditions to mature hepatocytes for liver tissue engineering. J. Tissue Eng. Regen. Med..

[87] Zambon A (2015). High temporal resolution detection of patient-specific glucose uptake from human ex vivo adipose tissue on-chip. Anal. Chem..

[88] Ju X, Li D, Gao N, Shi Q, Hou H (2008). Hepatogenic differentiation of mesenchymal stem cells using microfluidic chips. Biotechnol. J..

[89] Chao P, Maguire T, Novik E, Cheng KC, Yarmush ML (2009). Evaluation of a microfluidic based cell culture platform with primary human hepatocytes for the prediction of hepatic clearance in human. Biochem. Pharmacol..

[90] Imura Y, Asano Y, Sato K, Yoshimura E (2009). A microfluidic system to evaluate intestinal absorption. Analyt. Sci..

[91] Li Y, Qin J, Lin B, Zhang W (2010). The effects of insulin-like growth factor-1 and basic fibroblast growth factor on the proliferation of chondrocytes embedded in the collagen gel using an integrated microfluidic device. Tissue Eng. Part C Methods.

[92] An D, Kim K, Kim J (2014). Microfluidic system based high throughput drug screening system for curcumin/trail combinational chemotherapy in human prostate cancer PC3 cells. Biomol. Ther. (Seoul).

[93] Ju, S. M., Jang, H. J., Kim, K. B. & Kim, J. High-throughput cytotoxicity testing system of acetaminophen using a microfluidic device (MFD) in HepG2 cells. **78**, 1063–1072 (2015). 10.1080/15287394.2015.1068650 10.1080/15287394.2015.1068650 26241707

[94] Guild J (2016). Embryonic stem cells cultured in microfluidic chambers take control of their fate by producing endogenous signals including LIF. Stem Cells.

[95] Toh YC, Raja A, Yu H, Van Noort D (2018). A 3D microfluidic model to recapitulate cancer cell migration and invasion. Bioengineering.

[96] Fede C (2017). Influence of shear stress and size on viability of endothelial cells exposed to gold nanoparticles. J. Nanopart. Res..

[97] Prot JM (2011). Improvement of HepG2/C3a cell functions in a microfluidic biochip. Biotechnol. Bioeng..

[98] Sasaki N (2012). A palmtop-sized microfluidic cell culture system driven by a miniaturized infusion pump. Electrophoresis.

[99] Jang K-J (2013). Human kidney proximal tubule-on-a-chip for drug transport and nephrotoxicity assessment. Integr. Biol..

[100] Vereshchagina E, Mc Glade D, Glynn M, Ducrée J (2013). A hybrid microfluidic platform for cell-based assays via diffusive and convective trans-membrane perfusion. Biomicrofluidics.

[101] Hattori K (2014). Microfluidic perfusion culture chip providing different strengths of shear stress for analysis of vascular endothelial function. J. Biosci. Bioeng..

[102] Chi M (2015). A microfluidic cell culture device (μFCCD) to culture epithelial cells with physiological and morphological properties that mimic those of the human intestine. Biomed. Microdevices.

[103] Li J (2017). A microfluidic design to provide a stable and uniform in vitro microenvironment for cell culture inspired by the redundancy characteristic of leaf areoles. Lab. Chip.

[104] Tan SW (2019). Establishing a quick screening method by using a microfluidic chip to evaluate cytotoxicity of metal contaminants. Sci. Total Environ..

[105] Zhou Q (2015). Liver injury-on-a-chip: microfluidic co-cultures with integrated biosensors for monitoring liver cell signaling during injury. Lab. Chip.

[106] Suter-Dick L (2018). Combining extracellular miRNA determination with microfluidic 3D cell cultures for the assessment of nephrotoxicity: A proof of concept study. AAPS J..

[107] AhmadVaez S (2018). The cardiac niche role in cardiomyocyte differentiation of rat bone marrow-derived stromal cells: Comparison between static and microfluidic cell culture methods. Excli. J..

[108] Yu F (2020). A pump-free tricellular blood–brain barrier on-a-chip model to understand barrier property and evaluate drug response. Biotechnol. Bioeng..

[109] Lee-Montiel FT (2021). Integrated isogenic human induced pluripotent stem cell-based liver and heart microphysiological systems predict unsafe drug-drug interaction. Front. Pharmacol..

[110] Selmin G (2021). MYOD modified mRNA drives direct on-chip programming of human pluripotent stem cells into skeletal myocytes. Biochem. Biophys. Res. Commun..

[111] Giobbe GG (2015). Functional differentiation of human pluripotent stem cells on a chip. Nat. Methods..

[112] Jung DJ (2019). A one-stop microfluidic-based lung cancer organoid culture platform for testing drug sensitivity. Lab. Chip.

[113] Priyadarshani J, Roy T, Das S, Chakraborty S (2021). Frugal approach toward developing a biomimetic, microfluidic network-on-a-chip for in vitro analysis of microvascular physiology. ACS Biomater. Sci. Eng..

[114] Jaberi A (2020). Microfluidic systems with embedded cell culture chambers for high-throughput biological assays. ACS Appl. Bio Mater..

[115] Atif AR, Pujari-Palmer M, Tenje M, Mestres G (2021). A microfluidics-based method for culturing osteoblasts on biomimetic hydroxyapatite. Acta Biomater..

[116] Hosic S (2021). Rapid prototyping of multilayer microphysiological systems. ACS Biomater. Sci. Eng..

[117] Van Engeland NCA (2018). A biomimetic microfluidic model to study signalling between endothelial and vascular smooth muscle cells under hemodynamic conditions. Lab. Chip.

[118] Xu H (2018). Impact of flow shear stress on morphology of osteoblast-like IDG-SW3 cells. J. Bone Miner. Metab..

[119] Bahmaee H (2020). Design and evaluation of an osteogenesis-on-a-chip microfluidic device incorporating 3D cell culture. Front. Bioeng. Biotechnol..

[120] Yazdian Kashani S, Keshavarz Moraveji M, Bonakdar S (2021). Computational and experimental studies of a cell-imprinted-based integrated microfluidic device for biomedical applications. Sci. Rep..

[121] Jackson-Holmes EL, Schaefer AW, McDevitt TC, Lu H (2020). Microfluidic perfusion modulates growth and motor neuron differentiation of stem cell aggregates. Analyst.

[122] Hassell BA (2017). Human organ chip models recapitulate orthotopic lung cancer growth, therapeutic responses, and tumor dormancy in vitro. Cell Rep..

[123] Zhou, Q. *et al.* Lab on a Chip Liver injury-on-a-chip: Microfluidic co-cultures with integrated biosensors for monitoring liver cell signaling during injury. **15**, (2014). 10.1039/c5lc00874c 26480303

[124] Lanz HL (2017). Therapy response testing of breast cancer in a 3D high-throughput perfused microfluidic platform. BMC Cancer.

[125] Kongsuphol P (2019). In vitro micro-physiological model of the inflamed human adipose tissue for immune-metabolic analysis in type II diabetes. Sci. Rep..

[126] Kim JH (2019). A microfluidic chip embracing a nanofiber scaffold for 3D cell culture and real-time monitoring. Nanomaterials.

[127] Lin DSY, Rajasekar S, Marway MK, Zhang B (2021). From model system to therapy: Scalable production of perfusable vascularized liver spheroids in ‘open-top’ 384-well plate. ACS Biomater. Sci. Eng..

[128] Perottoni S (2021). Intracellular label-free detection of mesenchymal stem cell metabolism within a perivascular niche-on-a-chip. Lab. Chip.

[129] Fernandez, C. E. *et al.* Human vascular microphysiological system for in vitro drug screening. *Sci. Rep*. **6**, (2016). 10.1038/srep21579 PMC4757887 26888719

[130] Wang X, Lee J, Ali M, Kim J, Lacerda CMR (2017). Phenotype transformation of aortic valve interstitial cells due to applied shear stresses within a microfluidic chip. Ann. Biomed. Eng..

[131] Bavli D (2016). Real-time monitoring of metabolic function in liver-onchip microdevices tracks the dynamics of Mitochondrial dysfunction. Proc. Natl. Acad. Sci. USA.

[132] Wevers, N. R. *et al.* A perfused human blood-brain barrier on-a-chip for high-throughput assessment of barrier function and antibody transport. *Fluids Barriers CNS*. **15**, (2018). 10.1186/s12987-018-0108-3 PMC6117964 30165870

[133] Ragelle, H. *et al.* Human retinal microvasculature-on-a-chip for drug discovery. *Adv. Healthc. Mater*. **9**, (2020). 10.1002/adhm.202001531 32975047

[134] Lee HN (2021). Effect of biochemical and biomechanical factors on vascularization of kidney organoid-on-a-chip. Nano Converg..

[135] Zeller P (2017). Hepatocytes cocultured with Sertoli cells in bioreactor favors Sertoli barrier tightness in rat. J. Appl. Toxicol..

[136] Lin N (2020). Repeated dose multi-drug testing using a microfluidic chip-based coculture of human liver and kidney proximal tubules equivalents. Sci. Rep..

[137] Musah S (2017). Mature induced-pluripotent-stem-cell-derived human podocytes reconstitute kidney glomerular-capillary-wall function on a chip. Nat. Biomed. Eng..

[138] Guo Y, Deng P, Chen W, Li Z (2020). Modeling pharmacokinetic profiles for assessment of anti-cancer drug on a microfluidic system. Micromachines..

[139] Fois CAM, Schindeler A, Valtchev P, Dehghani F (2021). Dynamic flow and shear stress as key parameters for intestinal cells morphology and polarization in an organ-on-a-chip model. Biomed. Microdevices.

[140] Ross EJ (2021). Three dimensional modeling of biologically relevant fluid shear stress in human renal tubule cells mimics in vivo transcriptional profiles. Sci. Rep..

[141] Sato K, Sato M, Yokoyama M, Hirai M, Furuta A (2018). Influence of culture conditions on cell proliferation in a microfluidic channel. Analyt. Sci..

[142] Chen C (2018). Insert-based microfluidics for 3D cell culture with analysis. Anal. Bioanal. Chem..

[143] Altmann B (2014). Differences in morphogenesis of 3D cultured primary human osteoblasts under static and microfluidic growth conditions. Biomaterials.

[144] Oliveira NM (2017). Open fluidics: A cell culture flow system developed over wettability contrast-based chips. Adv. Healthc. Mater..

[145] Toh Y-C, Voldman J (2011). Fluid shear stress primes mouse embryonic stem cells for differentiation in a self-renewing environment via heparan sulfate proteoglycans transduction. FASEB J..

[146] Van Der Meer AD, Vermeul K, Poot AA, Feijen J, Vermes I (2010). Flow cytometric analysis of the uptake of low-density lipoprotein by endothelial cells in microfluidic channels. Cytometry A.

[147] Zheng W (2012). Fluid flow stress induced contraction and re-spread of mesenchymal stem cells: A microfluidic study. Integr. Biol..

[148] Rennert K (2015). A microfluidically perfused three dimensional human liver model. Biomaterials.

[149] Hasenberg T (2015). Emulating human microcapillaries in a multi-organ-chip platform. J. Biotechnol..

[150] Bruce A (2015). Three-dimensional microfluidic tri-culture model of the bone marrow microenvironment for study of acute lymphoblastic leukemia. PLoS ONE.

[151] Ishida T (2016). Investigation of the influence of glucose concentration on cancer cells by using a microfluidic gradient generator without the induction of large shear stress. Micromachines..

[152] Kongsuphol P, Liu Y, Ramadan Q (2016). On-chip immune cell activation and subsequent time-resolved magnetic bead-based cytokine detection. Biomed. Microdevices.

[153] Shao J (2009). Integrated microfluidic chip for endothelial cells culture and analysis exposed to a pulsatile and oscillatory shear stress. Lab. Chip.

[154] Buchanan CF (2014). Three-dimensional microfluidic collagen hydrogels for investigating flow-mediated tumor-endothelial signaling and vascular organization. Tissue Eng. Part. C Methods.

[155] Bricks T (2014). Development of a new microfluidic platform integrating co-cultures of intestinal and liver cell lines. Toxicol. In Vitro.

[156] Kondo E, Wada KI, Hosokawa K, Maeda M (2014). Microfluidic perfusion cell culture system confined in 35 mm culture dish for standard biological laboratories. J. Biosci. Bioeng..

[157] Abaci HE, Shen YI, Tan S, Gerecht S (2014). Recapitulating physiological and pathological shear stress and oxygen to model vasculature in health and disease. Sci. Rep..

[158] Huang K, Boerhan R, Liu C, Jiang G (2017). Nanoparticles Penetrate into the multicellular spheroid-on-chip: Effect of surface charge, protein corona, and exterior flow. Mol. Pharm..

[159] Kane BJ, Zinner MJ, Yarmush ML, Toner M (2006). Liver-specific functional studies in a microfluidic array of primary mammalian hepatocytes. Anal. Chem..

[160] Reinitz A, DeStefano J, Ye M, Wong AD, Searson PC (2015). Human brain microvascular endothelial cells resist elongation due to shear stress. Microvasc. Res..

[161] Kocal GC (2016). Dynamic microenvironment induces phenotypic plasticity of esophageal cancer cells under flow. Sci. Rep..

[162] Christoffersson J (2018). A cardiac cell outgrowth assay for evaluating drug compounds using a cardiac spheroid-on-a-chip device. Bioengineering.

[163] Kimura H (2018). Effect of fluid shear stress on in vitro cultured ureteric bud cells. Biomicrofluidics.

[164] Arora S, Lam AJY, Cheung C, Yim EKF, Toh YC (2019). Determination of critical shear stress for maturation of human pluripotent stem cell-derived endothelial cells towards an arterial subtype. Biotechnol. Bioeng..

[165] Yu F (2020). A vascular-liver chip for sensitive detection of nutraceutical metabolites from human pluripotent stem cell derivatives. Biomicrofluidics.

[166] Arik YB (2021). Microfluidic organ-on-a-chip model of the outer blood–retinal barrier with clinically relevant read-outs for tissue permeability and vascular structure. Lab Chip.

[167] Siren EMJ (2021). An improved in vitro model for studying the structural and functional properties of the endothelial glycocalyx in arteries, capillaries and veins. FASEB J..

[168] Mani V (2019). Epithelial-to-mesenchymal transition (EMT) and drug response in dynamic bioengineered lung cancer microenvironment. Adv. Biosyst..

[169] Tan K (2019). A high-throughput microfluidic microphysiological system (PREDICT-96) to recapitulate hepatocyte function in dynamic, re-circulating flow conditions. Lab Chip.

[170] Zheng Y (2019). Multifunctional regulation of 3D cell-laden microsphere culture on an integrated microfluidic device. Anal. Chem..

[171] Skafte-Pedersen, P. *et al.* A self-contained, programmable microfluidic cell culture system with real-time microscopy access. *Biomed. Microdevices*. **14**, (2012). 10.1007/s10544-011-9615-6 22160447

[172] Hemmingsen, M. *et al.* The role of paracrine and autocrine signaling in the early phase of adipogenic differentiation of adipose-derived stem cells. *PLoS One*. **8**, (2013). 10.1371/journal.pone.0063638 PMC3665830 23723991

[173] Martinez-Jimenez, C. P., Jover, R., Teresa Donato, M., Castell, J. V. & Jose Gomez-Lechon, M. Transcriptional Regulation and Expression of CYP3A4 in Hepatocytes. *Curr. Drug. Metab.* **8**, 185–194 (2007). 10.2174/138920007779815986 17305497

[174] Valkama AJ (2018). Optimization of lentiviral vector production for scale-up in fixed-bed bioreactor. Gene Ther..

