# Supplementary material for: A quantitative meta-analysis comparing cell models in perfused organ on a chip with static cell cultures
Source: Sci Rep. 2023 May 22;13:8233. doi: 10.1038/s41598-023-35043-5 (PMC10203308; doi:10.1038/s41598-023-35043-5)
Supplement: Supplementary file 1 — Supplementary Information. [file 41598_2023_35043_MOESM1_ESM.docx]

Supplementary figures


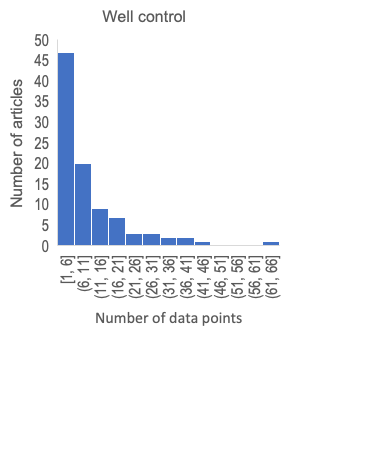

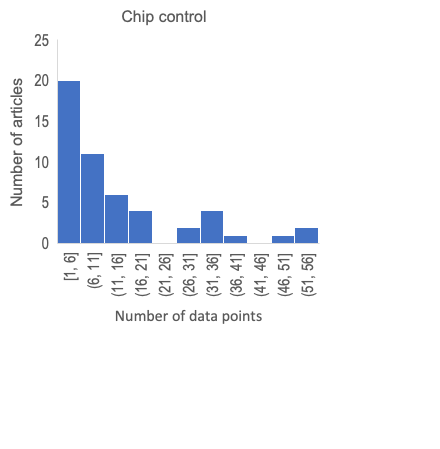


Supplementary figure 1. The distribution of ratio collected per article. The ratio is the same as an independent biomarker. Most articles only collect information from up to five independent biomarkers. The data was analysed from Supplementary table 2 and 3 respectively.


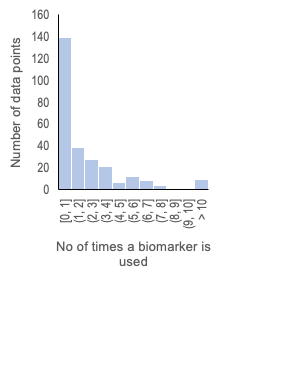


Supplementary figure 2. The distribution of the number of times a biomarker has been used. The graphs show often a biomarker is used. Most biomarkers are used only once in the literature. These are typically mRNA. Other are used by many articles such as viability >10 articles.

| 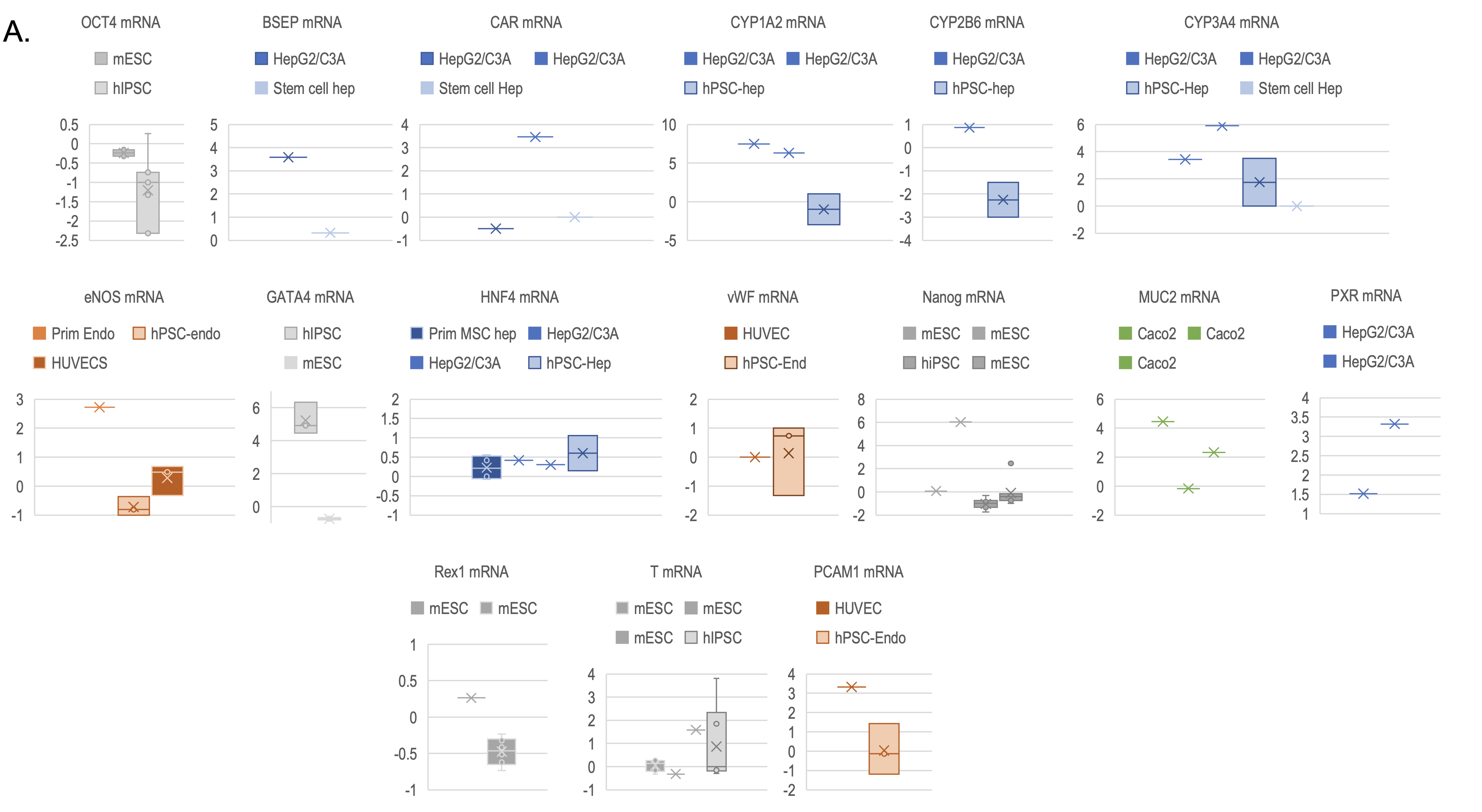  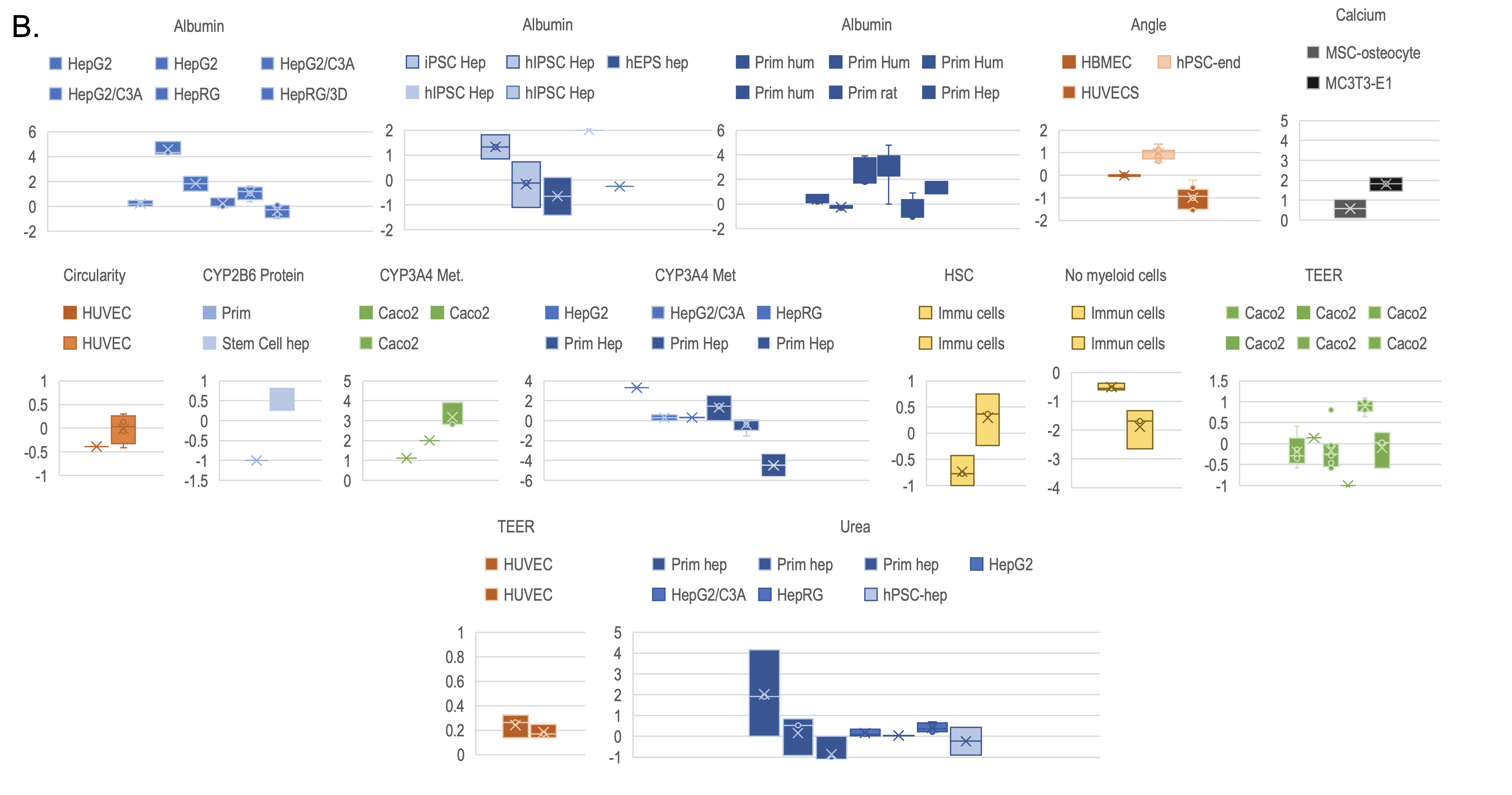 |
| --- |
| Supplementary figure 3. Log2 ratio boxplots per biomarker and tissue/cell type. The data between chips and wells as controls are fused in these graphs to obtain as many possible biomarkers as possible that were investigated in at least two articles. Each article is denoted with its own bar in the respective graph. A) mRNA data. B) all other biomarkers. |


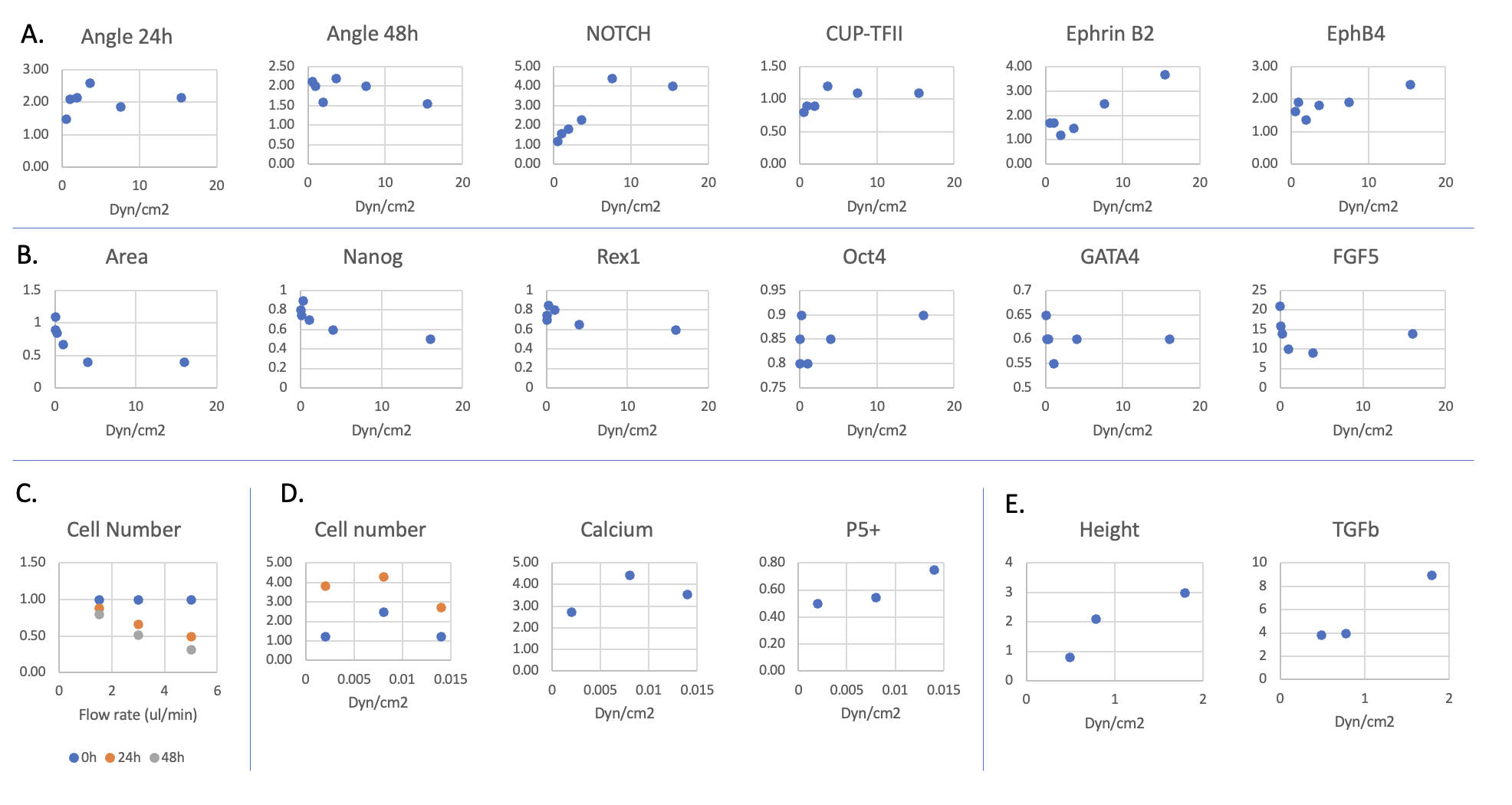


Supplementary figure 4. Ratio (linear scale) between dynamic incubation give different shear or having different flow rate. A. Vessels (endothelium) ^1^, B. Stem cells^2^, C. Cancer (Hela)^3^, D. Bone (osteoblasts)^4^ and E. aortic valvular interstitial cells^5.^

**References**:

1. Arora, S., Lam, A. J. Y., Cheung, C., Yim, E. K. F. & Toh, Y. C. Determination of critical shear stress for maturation of human pluripotent stem cell-derived endothelial cells towards an arterial subtype. *Biotechnol Bioeng* **116**, 1164–1175 (2019).

2. Toh, Y.-C. & Voldman, J. Fluid shear stress primes mouse embryonic stem cells for differentiation in a self-renewing environment via heparan sulfate proteoglycans transduction. *The FASEB Journal* **25**, 1208–1217 (2011).

3. Ishida, T. *et al.* Investigation of the Influence of Glucose Concentration on Cancer Cells by Using a Microfluidic Gradient Generator without the Induction of Large Shear Stress. *Micromachines 2016, Vol. 7, Page 155* **7**, 155 (2016).

4. Atif, A. R., Pujari-Palmer, M., Tenje, M. & Mestres, G. A microfluidics-based method for culturing osteoblasts on biomimetic hydroxyapatite. *Acta Biomater* **127**, 327–337 (2021).

5. Wang, X., Lee, J., Ali, M., Kim, J. & Lacerda, C. M. R. Phenotype Transformation of Aortic Valve Interstitial Cells Due to Applied Shear Stresses Within a Microfluidic Chip. *Ann Biomed Eng* **45**, 2269–2280 (2017).
